# The Effects of Transcutaneous Electrical Nerve Stimulation on Post-Episiotomy Pain Severity in Primiparous Women: A Randomized, Controlled, Placebo Clinical Trial

**DOI:** 10.31661/gmj.v8i0.1404

**Published:** 2019-08-14

**Authors:** Seyedeh Soma Zakariaee, Roonak Shahoei, Leila Hashemi Nosab, Ghobad Moradi, Mina Farshbaf

**Affiliations:** ^1^Faculty of Nursing and Midwifery, Kurdistan University of Medical Sciences, Sanandaj, Iran; ^2^Department of Epidemiology, Faculty of Medicine, Kurdistan University of Medical Sciences, Sanandaj, Iran; ^3^Department of Gynecology, Faculty of Medicine, Kurdistan University of Medical Sciences, Sanandaj, Iran

**Keywords:** Transcutaneous Electrical Nerve Stimulation, Primiparous, Episiotomy Pain, Postpartum Period

## Abstract

**Background:**

Episiotomy or cutting the perineum is the most common operation in obstetrics. Perineal pain is the main complication of episiotomy that affects the quality of life and mental health of the mother. Reducing the pain and side effects of the chemical drugs prescribed for pain relief has attracted the attention of both physicians and scientists. This study was conducted to determine the effects of transcutaneous electrical nerve stimulation (TENS), as an alternative method, on post-episiotomy pain severity.

**Materials and Methods:**

This randomized clinical trial was performed on 120 primiparous women who had referred to Sanandaj Besat Hospital in 2018. The patients were divided randomly into three groups, including the intervention group (TENS-On), the placebo group (TENS-Off), and the control group. TENS electrodes were placed near the episiotomy site in genitofemoral and pudendal nerves. The pain was measured after episiotomy in lying, sitting, and activity positions. The pain severity was measured using a pain measurement instrument (numeral rating score) at four-time points, i.e., before the intervention as well as 30, 60, and 120 minutes after the TENS intervention. For data analysis, Pearson correlation, student’s t-test, Kruskal-Wallis test, ANOVA test, and Mann-Whitney U tests were used.

**Results:**

Intragroup evaluation results for placebo and control groups demonstrated no significant difference in the pain score (P>0.05). A significant difference was observed between the mean pain severity of the intervention group and that of the group with walking activities (P=0.04). In terms of the intergroup evaluation, there was a significant difference observed between the mean pain severity of the lying position and that of the control group (P=0.008). Regarding the sitting position, no significant difference was observed between its mean pain severity and that of the other two groups (P=0.04).

**Conclusion:**

TENS is an effective and safe method for post-episiotomy pain relief and a routine method used in the obstetrics and gynecology ward.

## Introduction

Episiotomy during childbirth is the most common surgical operation in midwifery [1], which is used to facilitate delivery [2]. Although the best episiotomy technique and its clinical benefits remain by far unknown [3], this operation is currently used as ever [4]. At least 65% of women in developed countries and 35% to 45% of women in underdeveloped countries experience episiotomy [1]. The prevalence rate of episiotomy is higher in Asian women due to the differences in the anatomy and elasticity of their pelvic floor muscle than other races [5]. Over 80% of Iranian women with first and multiple gestation pregnancies might have experienced episiotomy [6]. In Iran, mediolateral episiotomy is more common than median episiotomy. Hence, the prevalence rate of complications in Iranian women is higher [7]. Like other surgical procedures, episiotomy has some complications, including pain, fever, vulva hematoma, rupture extension [8, 9], bleeding, infection, inflammation, edema, suture opening, and fistula [10-12]. The most common complication of episiotomy is the perineal pain [13], with the postnatal prevalence of 96.4% on the first day, 63% on the second day, and 25% on the 40^th^ day from childbirth [7]. Ten percent of women suffer from the perineal pain up to three months after delivery [14]. This complication affects physical, psychological, and social health of women [15, 16]. The effective treatment of this pain is quite important because of its unpleasant physiological and psychological outcomes from both economical and patients’ points of view [17]. Several methods have been suggested to reduce the perineal pain, including the use of medicinal products, such as aspirin-codeine, acetaminophen-codeine, nonsteroidal anti-inflammatory drugs [18], epidural analgesia, and the lidocaine gel. Besides, medicinal herbs, such as olives, lavender, aloe vera, chamomile, evergreen flowers, cinnamon [19], turmeric, pineapples [8], and non-medicinal methods, such as laser therapy, acupuncture, pelvic floor exercises, electrical stimulation [19], heat and cold treatments, relaxation methods, thought distraction, music therapy [2], and perineal massage [20] are effective in reducing the perineal pain. In recent years, the transcutaneous electrical nerve stimulation (TENS) method has been of great interest to researchers [21]. TENS is a non-medicinal, inexpensive, non-invasive, and safe method with minimal side effects that control pain by generating electrical pulses through the skin [22, 23]. The main mechanism of the TENS’ effect is still debatable, but its effectiveness can be explained using the gate control theory of pain and activation of the internal opioid system [24-28]. In other words, TENS leads to sensory stimulation and an impulse increase in large diameter fibers (A-beta), without stimulation in small diameter fibers (c) [21]. The effective range of TENS has been reported to be 30% to 66% [29]. In a study by Pitangui *et al*., the effects of high and low TENS frequencies were examined on pain relief after episiotomy. The results showed that high and low TENS frequencies were effective in relieving the post-episiotomy pain [30]. In a comparison made between the effects of TENS and lidocaine on the post-episiotomy pain, edema and tenderness of the perineum area, analgesic medication dosage, and the time needed for leaving the bed were significantly lower in the group with TENS than other groups. However, there was no significant difference in pain severity [31]. The comparison of the effects of TENS and the local infusion of lidocaine on episiotomy complications showed that the pain severity was significantly higher during the episiotomy repair phase in the group with TENS [32]. In the study by Lorenzana (1999), it was reported that TENS did not affect relieving the episiotomy-induced pain [33]. Although studies have demonstrated the effectiveness of TENS in many surgical procedures [34-40], no previous study in Iran has investigated the effects of TENS on the post-episiotomy pain via local application in the perineal area. Hence, given the importance of the post-episiotomy pain relief and the need for using an effective and non-invasive method, this study was conducted to determine the effectiveness of TENS as a pain relief source applied to primiparous women.

## Materials and Methods

This study was a randomized, controlled, placebo clinical trial conducted from April 2018 to June 2018 in the maternity ward of Sanandaj Besat Hospital, a training hospital and a referral center for the obstetric and nursing care in the Kurdistan province, Iran.

### Patients

The studied samples consisted of 120 primiparous puerperal women who had experienced spontaneous vaginal delivery with mediolateral episiotomy.

### Sample Size Calculation

For each group, the population sample size was measured at 37 based on the Cochran formula, with the power value of 0.80, alpha value of 0.05, and the standard deviation value of 0.78. To improve the power of the study, the sample size of 40 was considered for each group. The patients were divided into three groups by block randomization, including the intervention group (TENS-ON), the placebo group (TENS-OFF), and the control group (routine care without using TENS).

$$n\geq \frac{{\left(Z_{1-\frac{\alpha }{2}}+Z_{1-\beta }\right)}^{2}+(\sigma 1^{2}+\frac{\sigma 2^{2}}{2})}{{\left(\mu 1-\mu 2\right)}^{2}}$$

### Inclusion and Exclusion Criteria

The inclusion criteria of the study were primiparous women, aged over 18, the experience of low-risk pregnancy and singleton pregnancy, with a live newborn, the gestational age of between 38-42 weeks, cephalic and anterior occiput positions, the newborns weight ranged 2500-4000 g, the spontaneous vaginal delivery with mediolateral episiotomy, the experience of pain in the episiotomy site, no genitourinary pathology, having no drug addiction, no use of the sedative 4-6 hours before the intervention, and no use of medications during the data collection period. The exclusion criteria included the use of pacemakers, arrhythmia, epilepsy, and mental health problems, epidural anesthesia, puerperal complications, previous exposures to TENS, and the complication occurrence due to TENS (burns, ulcers, and skin allergies).

### Study Groups

**Figure 1:**
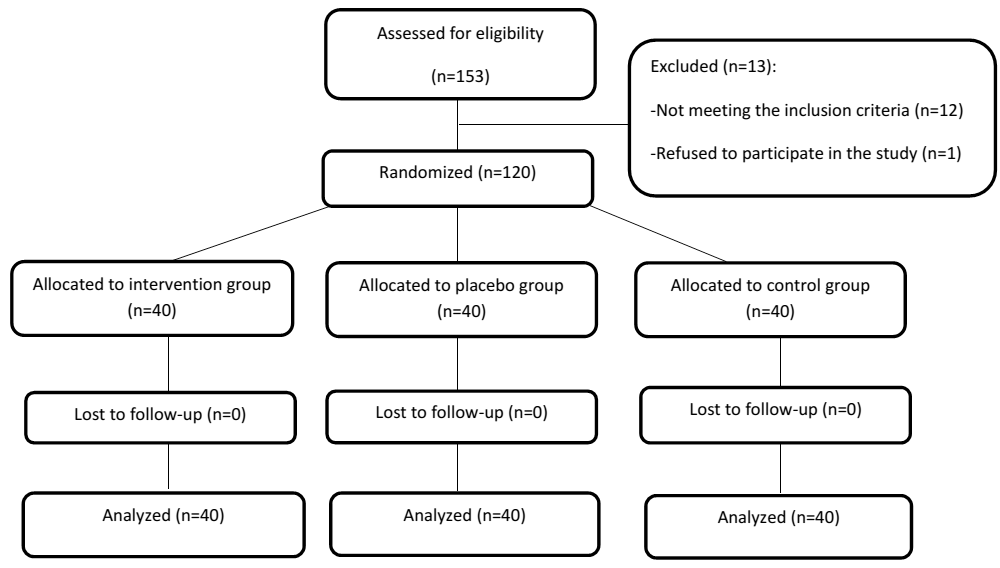
CONSORT flowchart of the study

**Figure 2:**
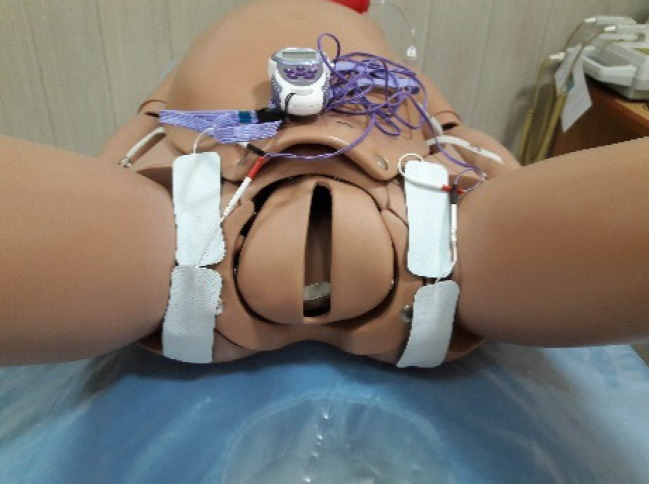
Schematic representation of the positioning of the electrodes on the puerpere.

The data were collected using a demographic data form with the variables of age, education level, job, residency, ethnicity, marital satisfaction, satisfaction with the newborn’s gender, mother’s exercise status, and mother’s body mas index (BMI), maternal clinical features (include the type of pregnancy, the gestational age, number of pregnancies, number of abortions, participation in physiological delivery classes, perineal massage during pregnancy, mother’s vital signs before and after the intervention), delivery features (the duration of the first stage of labor, duration of the second stage of labor, duration of the third stage of labor, incidence of shoulder dystocia during delivery), infant features (newborn’s gender, weight, and head circumference), a registration form for the pain (pain severity before the intervention in lying, sitting, and activity positions, pain severity 30 minutes after the intervention in lying, sitting, and activity positions, pain severity 60 minutes after the intervention in lying, sitting, and activity positions, as well as pain severity 120 minutes after the intervention in lying, sitting, and activity positions), together with the TENS’ complications and satisfaction rate. A numerical rating scale was used to measure the pain severity, i.e., a 10cm line using that people marked their pain intensity or indicate their pain by a number. In this approach, 0 corresponded to no pain and 10 represented the maximum pain ever experienced. The pain scale was divided into three groups, where 1-3, 4-7, and 8-10 were defined as mild, moderate, and severe pains, respectively. All eligible primiparous women were divided randomly into three groups by closed envelopes (Figure-1). The study began 6-24 hours after delivery (six hours after delivery, the mothers were advised to leave the bed and perform activities). The limitation of 24 hours was considered, since the acute period of the pain and inflammation occurs during this period, and the TENS treatment was conducted using a portable appliance model (Elle Tens made by Babycare Company, London) that generated a balanced pulse of biphasic and asymmetric waves with control switches for the frequency and amplitude variation. Four silicone-carbon electrodes were placed on the skin parallel to the episiotomy site (Figure-2). The site was associated with the pudendal and genitofemoral nerves that supply the perineal area. In the intervention group, TENS was conducted for 60 minutes at a frequency of 100 Hz and 75µSec pulse. In the placebo group, TENS electrodes were placed in the same places, but the device was turned off. In the control group, only the routine care was provided to the women in the postpartum period, and the electrodes were not placed. For each of the three groups, pain severity was evaluated in both lying and activity positions (including sitting and walking), 30 minutes, 60 minutes, and 120 minutes after TENS.

### Ethical Statement

This study was registered at the Iranian Center for Clinical Trials under code “IRCT20171224038034N1” after the approval of the research project in the Ethics Committee of Kurdistan University of Medical Sciences (ethics code: IR.MUK.REC.1396/365). Informed consent was obtained from the participants for cooperation in this study and publishing their data. The participants were informed that they could discontinue the interviews at any time.

### Statistical Analysis

The data analysis was conducted using SPSS Statistics version 20 (IBM, Illinois, Chicago, USA). For data description, the mean, standard deviation, and relative frequency were used. For data analysis, Pearson correlation, student’s t-test, Kruskal-Wallis test, analysis of variance test, and Mann-Whitney U test were utilized. A P<0.05 was considered as significant difference.

## Results

The participants’ features were not different among the groups (P>0.05). The baseline features of the participants are presented in Tables-1 and 2. The mean pain severity before the intervention was similar in the three groups of lying (P=0.50), sitting (P=0.20), and activity (P=0.30) positions, with no significant difference ( Table-3). The results showed that in the intervention group in the lying position, the pain score after the intervention in the second, third and fourth assessments were 6.18, 3.18, and 3.36, respectively. In the intervention group in the activity position, the pain score after the intervention in the second, third, and fourth assessments were 3.62, 1.72, and 3.20, respectively. In the intervention group in the sitting position, the pain score after the intervention in the second, third, and fourth assessments decreased (5.50, 5, 5). In the placebo and control groups, there was no significant difference in the pain score for any of the evaluations. Regarding pain severity 30 minutes after the intervention, the results showed no significant difference among the three groups. Besides, 60 minutes after the intervention, there was a considerable difference in the pain severity for lying and activity positions. The mean pain severity 60 minutes after the intervention, in the intervention group in lying and activity positions, have significant differences, yet there was no significant difference in the pain score for the three groups in the sitting position. For the three groups, 120 minutes after the intervention, there was no significant difference in the mean pain severity. Also, the mean pain severity for the intervention and placebo groups in the lying position was significantly different between the first and second assessments, as well as the third and fourth assessments (Table-4).

**Table 1:** Demographic Characteristics of Participants

**Variables**		** Intervention group (TENS-ON ) **	** Placebo group (TENS-OFF) **	** Control group(Routine care ) **	**P-value**	**Test**
**Age**	≤20	9 (22.50)	7 (17.50)	7 (17.50)	0.49	Kruskal-Wallis
	21-25	15 (37.50)	20 (50.00)	18 (45.00)		
	26—30	13 (32.50)	9 (22.50)	13 (32.50)		
	≥31	3 (7.50)	4 (10.00)	2 (5.00)		
**Education level**	Primary school education	6 (15.00)	7 (17.50)	5 (12.50)	0.25	Independent T-test
	Secondary school education	7 (17.50)	8 (20.00)	6 (15.00)		
	High school	8 (20.00)	5 (12.50)	7 (17.50)		
	High school diploma	12 (30.00)	15 (37.50)	13 (32.50)		
	Above	7 (17.50)	5 (12.50)	9 (22.50)		
**Job status**	Householder	30 (75.00)	30 (75.00)	30 (75.00)	0.44	Independent T-test
	Government employee	10 (25.00)	10 (25.00)	10 (25.00)		
**Residency**	Urban	27 (67.50)	26 (65.00)	33 (82.50)	0.17	Pearson correlation
	Rural	13 (32.50)	14 (35.00)	7 (17.50)		
**Ethnicity**	Kurdish	40 (100.00)	39 (97.50)	39 (97.50)	>0.99	Independent T-test
	Turkish	0 (0.00)	0 (0.00)	0 (0.00)		
	Fars	0 (0.00)	1 (2.50)	1 (2.50)		
	Lore	0 (0.00)	0 (0.00)	0 (0.00)		
**Marital satisfaction**	Yes	37 (92.50)	39 (97.50)	38 (95.00)	0.87	Independent T-test
	No	3 (7.50)	1 (2.50)	2 (5.00)		
**BMI**	Mean ± SD	28.83 ± 3.73	27.53 ± 3.64	28 ± 2.20	0.45	Kruskal-Wallis
**Satisfaction about the newborn’s sex**	Yes	35 (87.50)	38 (95.00)	33 (82.50)	0.25	Independent T-test
	No	5 (12.50)	2 (5.00)	7 (17.50)		
**Mother’s exercise status**	Yes	11 (27.50)	8 (20.00)	8 (20.00)	0.65	Pearson correlation
	No	29 (72.50)	32 (80.00)	32 (80.00)		

**BMI:** Body mass index

**Table 2:** Maternal Clinical and Newborn Characteristics of Participants

**Variables**		** Intervention group (TENS-ON ) **	** Placebo group (TENS-OFF) **	** Control group (Routine care) **	**P-value**	**Test**
**Type of Pregnancy**	Wanted	29 (72.50)	35 (87.50)	32 (80.00)	0.26	Independent T-test
	Unwanted	11 (27.50)	5 (12.50)	8 (20.00)		
**Gestational Age (week)**	Mean ± SD	39.3 ± 1.12	39.2 ± 1.13	39.5 ± 1.13	0.85	ANOVA
**Number of pregnancies**	1	35 (87.50)	32 (80.00)	34 (85.00)	0.92	Independent T-test
	2	3 (7.50)	5 (12.50)	3 (7.50)		
	3	2 (5.00)	3 (7.50)	3 (7.50)		
**Number of abortions**	0	35 (87.50)	32 (80.00)	34 (85.00)	0.92	Independent T-test
	1	3 (7.50)	5 (12.50)	3 (7.50)		
	2	2 (5.00)	3 (7.50)	3 (7.50)		
**Participation in the physiological delivery classes**	Yes	20 (50.00)	22 (55.00)	30 (75.00)	0.054	Pearson correlation
	No	20 (50.00)	18 (45.00)	10 (25.00)		
**Perineal massage during pregnancy**	Yes	10 (25.00)	10 (25.00)	10 (25.00)	>0.99	Pearson correlation
	No	30 (75.00)	30 (75.00)	30 (75.00)		
**Duration of the first stage of Labor (hour)**	Mean ± SD	9.825 ± 3.43	10.375 ± 3.70	9.5 ± 2.276	0.55	Kruskal-Wallis
**Duration of the second stage of Labor (min)**	Mean ± SD	47.50 ± 25.54	49.50± 29.26	35.375 ± 14.69	0.057	Kruskal-Wallis
**Duration of the third stage of Labor (min)**	Mean ± SD	8.65 ± 5.18	8.875 ± 3.90	8.40 ± 7.71	0.31	Kruskal-Wallis
**Incidence of shoulder dystocia during delivery**	Yes	0 (0.00)	2 (5.00)	0 (0.00)	0.13	Pearson correlation
	No	40 (100.00)	38 (95.00)	40 (100.00)		
**Newborn’s sex**	Female	21 (52.50)	18 (45.00)	22 (55.00)	0.64	Pearson correlation
	Male	19 (47.50)	22 (55.00)	18 (45.00)		
**Newborn’s weight (gr)**	Mean ± SD	3247.75 ± 305.500	3306 ± 299.622	3308.75 ± 228.96	0.54	ANOVA
**Circumference of the baby’s head (cm)**	Mean ± SD	34.52 ± 1.52	34.7 ± 1.31	34.7 ± 1.32	0.78	ANOVA

**ANOVA:** Analysis of variance

**Table 3:** Comparison of Pain Severity before the Intervention in Three Groups

**Evaluations**	** Intervention group (TENS-ON ) Mean ± SD **	** Placebo group (TENS-OFF) Mean ± SD **	** Control group (Routine care ) Mean ± SD **	**P-value**
**Resting status**	6.81 ± 1.60	6.08 ± 1.88	6.77 ± 1.78	0.504
**Sitting status**	6.07 ± 1.61	5.62 ± 1.51	5.40 ± 1.90	0.205
**Walking status**	4.32 ± 1.73	3.72 ± 1.30	3.90 ± 2.19	0.304

**Table 4:** Comparison of Pain Severity after the Intervention in Three Groups

**Evaluations after intervention**	** Intervention group (TENS-ON ) Mean ± SD **	** Placebo group (TENS-OFF) Mean ± SD **	** Control group (Routine care ) Mean ± SD **	**P-value**
**30 minute**				
Resting status	6.18 ± 2.31	5.66 ± 2.57	5.44 ± 1.87	0.695
Sitting status	5.50 ± 1.62	5.60 ± 1.50	5.60 ± 1.89	0.931
Walking status	3.62 ± 1.53	3.45 ± 1.41	3.95 ± 2.16	0.636
**60 minute**				
Resting status	3.18 ± 2.04	4.75 ± 1.65	6.44 ± 2.18	0.008
Sitting status	5 ± 1.97	5.57 ± 1.57	5.55 ± 1.96	0.167
Walking status	1.72 ± 2.19	3.26 ± 1.60	3.88 ± 2.08	0.046
**120 minute**				
Resting status	3.36 ± 2.33	4.83 ± 2.16	5.22 ± 1.87	0.208
Sitting status	5 ± 1.97	5.42 ± 1.75	5.67 ± 1.99	0.282
Walking status	3.20 ± 1.52	3.12 ± 1.65	3.82 ± 2.10	0.174

## Discussion

The purpose of this study was to determine the effects of TENS on post-episiotomy pain severity in primiparous women with mediolateral episiotomy. Our results showed that TENS reduced the clinical pain in the intervention group immediately and one hour after the intervention. Other studies have also demonstrated TENS’ effectiveness in relieving postoperative pains in the cases of endometrial biopsy, uterine tubal ligation, inguinal herniorrhaphy, abortion, hysteroscopy, and primary dysmenorrhea [34-39]. In a study by Chiu *et al*. (1999), the effects of TENS were evaluated on pain severity after hemorrhoidectomy. The results showed that pain severity decreased significantly in the intervention group, 8, 12, 16, and 24 hours after the surgery [40]. In the TENS method, the pain relief mechanism is based on the gate control theory and the increase in the endorphins and enkephalins in the central nervous system [41]. In the intervention group, there was a significant difference in the mean pain severity in lying and activity positions in the third assessment (60 minutes after TENS), which showed a decrease in the pain severity in the intervention group. This result is in contradiction with the results of the study by Pitangui *et al*. [30]. They showed that the effects of high and low-frequency TENS on pain severity after episiotomy were evaluated. The mean pain severity decreased significantly, immediately, 30 minutes, and 60 minutes after the intervention in the high and low-frequency TENS groups in the lying position [30]. There was no significant difference in the case of the activity position. The inconsistencies observed could have been due to the differences in culture and race, as well as the frequency and duration of TENS [30] in the mentioned study, TENS electrodes were placed in the pudendal and genitofemoral nerves. The high-frequency TENS (100 Hz and a pulse of 100 µSec), the low-frequency TENS (5 Hz and a pulse of 100 µSec), and the placebo (TENS-Off) groups received stimulation for 30 minutes after the repair of episiotomy [30]. In another study, high-frequency TENS was used to relieve post-episiotomy pain [42]. A significant decrease was observed in the pain severity in the intervention group, in lying, sitting and activity positions, 60 and 120 minutes after TENS [42]. In the present study, there was no significant reduction observed in the mean pain severity in the assessments, in the sitting position. The differences between Pitangui *et al*. [42] findings and those of the current study could have been due to the remaining effects of epidural analgesia during labor. However, in the current study, the patients with epidural analgesia during labor were excluded to control the effect of this confounding factor. In the present study, a significant difference was observed in the pain score for the three groups in the walking position, similar to previous studies that reported a pain reduction at the time of walking, respiratory movements, and position changes [27, 42]. The location of the electrodes, the intervention time, the stimulation duration, as well as the frequency and intensity of the stimulation must be standardized to achieve the best results in TENS [23, 43]. In the current study, the electrodes were placed near the episiotomy site in the parallel mode, since there were pudendal and genitofemoral nerves that caused pain relief and general numbness in the perineum area. The perineal branch of the pudendal nerve tingling the muscles of the urogenital triangle and the skin of the large lobe. The genital branch of the genitofemoral nerve runs through to the skin of the anterior perineum, and the femoral branch of the nerve pins a part of the thigh [44]. In some studies, TENS electrodes were placed at the site of these nerves to reduce pain severity after episiotomy [30, 42]. In similar studies, TENS electrodes were placed near the surgical site to investigate TENS effects on the postoperative pain [45]. In some other studies that examined the effects of TENS on pain severity during episiotomy, electrodes were placed at the site of Hugo and Shen Men acupuncture points [31-33]. In the study by Rezaeyan *et al*. [32], the effects of TENS and local lidocaine injection on episiotomy complications were compared. The results showed that the pain severity was lower for the TENS group immediately as well as 1, 6, and 12 hours after the completion of the episiotomy repair. However, pain severity in the TENS group was greater during the episiotomy repair [32]. In the study by Lorenzana (1999), it was reported that TENS did not decrease the post-episiotomy pain by stimulating the Hugo and Shen Men points [33]. In this study, no side effect was seen during the TENS intervention, including skin sensitivity, ulcers, and burns. Some other studies have also reported no complication for TENS [36, 42, 46]. TENS has no side effects, i.e., the only side effects that may appear in its long-term use are skin irritation and allergy [38]. At the end of the intervention, the participants were asked about the use of TENS. All participants considered TENS as an appropriate device and demanded to re-use TENS to relieve their pain in the next delivery. In the study by Erdogan *et al*. [47], the effects of TENS were evaluated on the pain after tracheostomy and the pulmonary function. In that study, there was no report of dissatisfaction with TENS [47]. Participants in the placebo group also expressed their satisfaction with the use of TENS, with their satisfaction, could have been due to the receiving of the extra care and attention from the staff and researchers. The strengths of the current study included the basic evaluation of pain severity before the intervention, the random allocation of subjects, and the assessment of satisfaction with the side effects of the TENS intervention. The limitations of this study included the individual and genetic differences in the perception of pain severity, which was controlled as much as possible by random sampling. This study was the first clinical trial that investigated the effects of the electrical stimulation of genitofemoral and pudendal nerves through the skin on post-episiotomy pain severity in Iran.

## Conclusion

The results of the present study indicate that TENS is a safe, uncomplicated, comfortable, and effective pain reliever with a high rate of acceptability. To improve maternal care, this method could be used as a routine approach in the maternity ward to reduce the frequency of the postoperative pain after episiotomy.

## Acknowledgment

The present work has been extracted from the MSc thesis of Seyedeh Soma Zakariaee (thesis number: 1397.056), at the Department of Midwifery, School of Nursing and Midwifery, Kurdistan University of Medical Sciences. The authors would like to thank the Research Affairs Department of Kurdistan University of Medical Sciences, School of Nursing and Midwifery, Department of Nursing and Midwifery, the medical staff of Sanandaj Besat Hospital, as well as the participants.

## Conflict of Interest

The authors declare no conflict of interest and approve the final article.

## References

[1] Delaram M, Dadkhah N, Jafarzadeh L (2015). Comparing the effect of indomethacin suppository and mefenamic acid capsule on post episiotomy pain. IJNMR.

[2] Delaram M, Dadkhah N (2014). Comparing the Effects of Lidocaine Cream and Mefenamic Acid on Post Episiotomy Pain. IJOGI.

[3] Khajavi Shojae K, Dawati A, Zayeri F (2010). Persistent perineal pain after episiotomy in nulliparous women admitted to hospital in Tehran. JMSG.

[4] Pazandeh F, Savadzadeh S, Mojab F, Alavi Majd H (2008). Effect of Chamomile essence on episiotomy healing in primiparous women. J Ardabil Univ Med Sci.

[5] Amani R, Kariman N, Mojab F, Alavi Majd H, Majidi S (2015). Assessing comparison the effect of cooling gel pads and topical olive oil on the intensity of episiotomy pain in primiparous women. Complementary Medicine Journal.

[6] Khani S, Taringo F, Shabani B (2000). Episiotomy is protective of laceration genital system. Journal of modares med.

[7] Shojaei KK, Davati A, Zayeri F (2009). Frequency and side effect of episiotomy in primiparous women: a three month longitudinal survey. Qom Univ Med Sci J.

[8] Taleb S, Ozgoli G, Mojab F, Nsiri M, Ahvazi M (2016). Effect of Verbascum Thapsus cream on intensity of episiotomy pain in primiparous women. IJOGI.

[9] Dutta DC. Text book of obstetrics. 6th ed. Calcutta: New Central Book Agency; 2006. P. 568-71.

[10] Ahmadi Z (2015). Review of effective methods to reduce damage to the perineum during delivery and its recovery. IJOGI.

[11] Tara F, Golmakani N, Motlagh ER, Assili J, Shakeri M (2009). The effects of turmeric (Curcuma Longa L) ointment on healing of episiotomy site in Primiparous women. Int J Gynecol Obstet.

[12] Wilson PD, Herbison RM, Herbison GP (1996). Obstetrics and Gynecology. Int J Obstet Gynaecol.

[13] Islam A, Hanif A, Ehsan A, Arif S, Niazi SK, Niazi AK (2013). Morbidity from episiotomy. J Pak Med Assoc.

[14] Fauconnier A, Goltzene A, Issartel F, Janse-Marec J, Blondel B, Fritel X (2012). Late post-partum dyspareunia: Does delivery play a role?. Prog Urol.

[15] Kalichman L (2008). Perineal massage to prevent perineal trauma in childbirth. Isr Med Assoc J.

[16] Fernando RJ (2007). Risk factors and management of obstetric perineal injury. Obstet Gynaecol Reproduct Med.

[17] Swindale J (1989). The Nurses role in giving preoperative information to reduce anxiety in patients admitted at hospital for elective minor surgery. Journal of Advanced Nursing.

[18] Minassian V, Jazayeri A, Prien S, Timmons R, Stumbo K (2002). Randomized trial of lidocaine ointment versus placebo for the treatment of postpartum perineal pain. Obstet Gynecol.

[19] Shahrahmani H, Kariman N, Jannesari S, Ghalandari S, Asadi N (2016). A systematic review on the type of treatment methods to reduce pain and improve wound healing in Iran. IJOGI.

[20] Piri galledar A, Danesh kojori M, Jamshidi Manesh M, Hosseini F (2012). The effect of perineal massage on labor second stage period perineal tear and its outcomes. Journal of Shahrekord University of Medical Sciences.

[21] Dowswell T, Bedwell C, Lavender T, Neilson JP. Transcutaneous electrical stimulation for pain management in labor. Cochrane Database of Systematics Reviews.2009.Issues 2. Art No: CD007214. 10.1002/14651858.CD007214.pub2 PMC4297467 19370680

[22] Melzak R, Wall PD (1965). Pain mechanisms: A new theory. Science.

[23] Walsh DM, Home TE, Johnson MI, Sluka KA. Transcutaneous electrical nerve stimulation for acute pain. Cochrane Database of Systematics Reviews. 2009. 10.1002/14651858.CD006142.pub2 19370629

[24] Sluka KA, Walsh D (2003). Transcutaneous electrical nerve stimulation: Basic science mechanisms and clinical effectiveness. J Pain.

[25] Radhakrishnan R, Sluka KA (2005). Deep tissue afferents, but no cutaneous afferents, mediate transcutaneous electrical nerve stimulation-Indused antihyperalgesia. J Pain0.

[26] Sluka KA, Lisi TL, Westlund KN (2006). Increased release of serotonin in the spinal cord during low, but no high frequency transcutaneous electrical nerve stimulation in rats with joint inflammation. Arch Phys Med Rehabil.

[27] Rakel B, Frantz R (2003). Effectiveness of transcutaneous electrical nerve stimulation on postoperative pain with movement. J Pain.

[28] Breit R, Van der Wall H (2004). Transcutaneous electrical nerve stimulation for postoperative painrelief after total knee arthroplasty. J arthroplasty.

[29] Schofield P. Beyond pain. Phildephia: whurr;2006.

[30] Pitangui A, Araujo R, Bezerra M, Ribeiro C, Nakano AMS (2014). Low and high-frequency TENS in post-episiotomy pain relief: a randomized, double-blind clinical trial. J Phys Ther.

[31] Khedri P. Comparison of transcutaneous electrical nerve stimulation and lidocaine on episiotomy pain.[The Master Degree Thessis]. Ahvaz Jondishapur University of Medical Sciences. 2005.[in Persian].

[32] Rezaeyan M, Geranmayeh M, Direkvand-Moghadam A (2017). Comparison of transcutaneous electrical nerve stimulation and lidocaine on episiotomy complication in primiparous women. Iranian J nursing Midwifery Res.

[33] Lorenzana F (1999). A randomized controlled trial of the efficacy of transcutaneous electrical nerve stimulation(TENS) versus lidocaine in the relief of episiotomy pain. Philipp Journal Obestet Gynecol.

[34] De Angelis C, Perrone G, Santoro G, Nofroni I, Zichella L (2003). Suppression of pelvic pain during hysteroscopy with a transcutaneous electrical nerve stimulation device. Fertil Steril.

[35] Desantana JM, Sluka KA, Lauretti GR (2009). High and low frequency TENS reduce postoperative pain intensity after laparoscopic tubal ligation: a randomized controlled trial. Clin J Pain.

[36] Desantana JM, Santana-Filho VJ, Guerra DR, Sluka KA, Gurgel RQ, Da Silva VM Jr (2008). Hypoalgesic effect of the transcutaneous electrical nerve stimlation following inguinal herniorrhaphy: a randomized, controlled trial. J Pain.

[37] Bai H, Bai H, Yang Z (2017). Effect of transcutaneous electrical nerve stimulation therapy for the treatment of primary dysmenorrheal. Medicine.

[38] Platon B, Andrell P, Raner C, Rudolph M, Dvorestsky A, Mannheimer C (2010). High-frequency, high-intensity transcutaneous electrical nerve stimlation as treatment of pain after surgical abortion. Pain.

[39] Yilmazer M, Kose S, Arioz DT, Koken G, Ozbulut O (2012). Efficacy of transcutaneous electrical nerve stimulation for pain relief in women undergoing office endometrial biopsy. Arch Gynecol Obstet.

[40] Chiu JH, Chen WS, Chen CH, Jiang JK, Tang GJ (1999). Effect of transcutaneous electrical nerve stimulation for pain relief on patients undergoing hemorrhoidectomy: prospective, randomized, controlled trial. Dis Colon Rectum.

[41] Aghamohammadi A, Zafari M, Moslemi L (2009). The effect of Transcutaneous electrical nerve stimulation in acupuncture points on labor pain reduction. Babol University of Medical Sciences.

[42] Pitangui A, Sousa L, Gomes F, Ferreira C, Nakano A (2012). High-frequency TENS in post-episiotomy pain relief in primiparouspuerperae: a randomized, controlled trial. J Obstet Gynaecol Ress.

[43] Chesterton LS, Foster NE, Wright CC, Baxter GD, Barles P (2003). Effect of TENS frequency, intensity and stimulation site parameter manipulation on pressure pain thresholds in healthy human subjects. Pain.

[44] Richard S. Clinical anatomy.8th Ed: Wolters Kluwer;2004.

[45] Houshyar AE, Rezaie HH, Jahani Y, Kazemi M, Monfared S (2015). Comparison of two methods of aromatherapy with lavender essence and Transcutaneous Electrical Nerve Stimulation (TENS) on cesarean postoperative pain. IJOGI.

[46] Bjordal JM, Johnson MI, Ljunggreen AE (2003). Transcutaneous electrical nerve stimlation (TENS) can reduce postoperative analgesic consumption A meta-analysis with assessment of optimal treatment parameters for postoperative pain. Eur J Pain.

[47] Erdogan M, Erdogan A, Erbil N, Karakaya HK, Demircan A (2005). Postoperative, randomized, placebo-controlled study of the effect of TENS on postthoracotomy pain and pulmonary function. World J Surg.

